# Inflammation and neuronal plasticity: a link between childhood trauma and depression pathogenesis

**DOI:** 10.3389/fncel.2015.00040

**Published:** 2015-03-31

**Authors:** Annamaria Cattaneo, Flavia Macchi, Giona Plazzotta, Begni Veronica, Luisella Bocchio-Chiavetto, Marco Andrea Riva, Carmine Maria Pariante

**Affiliations:** ^1^Stress, Psychiatry and Immunology Laboratory, Department of Psychological Medicine, Institute of Psychiatry, King’s College LondonLondon, UK; ^2^IRCCS Centro S Giovanni di Dio, FatebenefratelliBrescia, Italy; ^3^Department of Pharmacological and Biomolecular Sciences, University of MilanMilan, Italy; ^4^Faculty of Psychology, eCampus UniversityNovedrate (Como), Italy

**Keywords:** childhood trauma, inflammation, stress, depression, neuroplasticity

## Abstract

During the past two decades, there has been increasing interest in understanding and characterizing the role of inflammation in major depressive disorder (MDD). Indeed, several are the evidences linking alterations in the inflammatory system to Major Depression, including the presence of elevated levels of pro-inflammatory cytokines, together with other mediators of inflammation. However, it is still not clear whether inflammation represents a cause or whether other factors related to depression result in these immunological effects. Regardless, exposure to early life stressful events, which represent a vulnerability factor for the development of psychiatric disorders, act through the modulation of inflammatory responses, but also of neuroplastic mechanisms over the entire life span. Indeed, early life stressful events can cause, possibly through epigenetic changes that persist over time, up to adulthood. Such alterations may concur to increase the vulnerability to develop psychopathologies. In this review we will discuss the role of inflammation and neuronal plasticity as relevant processes underlying depression development. Moreover, we will discuss the role of epigenetics in inducing alterations in inflammation-immune systems as well as dysfunction in neuronal plasticity, thus contributing to the long-lasting negative effects of stressful life events early in life and the consequent enhanced risk for depression. Finally we will provide an overview on the potential role of inflammatory system to aid diagnosis, predict treatment response, enhance treatment matching, and prevent the onset or relapse of Major Depression.

## Background

Major depressive disorder (MDD) is a highly prevalent complex neuropsychiatric condition characterized by a broad range of symptoms, which causes significant distress as well as impairment of normal functioning and that should not be attributable to a recent loss or to a general medical condition (American Psychiatric Association, 2000). Beside the classical monoaminergic hypothesis of depression, at least two major hypotheses have emerged based on dysfunction in immune-inflammatory systems (cytokine hypothesis) or in neuronal plasticity (neurotrophic hypothesis) (Schiepers et al., 2005; Calabrese et al., 2009; Maes et al., 2009; Miller et al., 2009; Castrén, 2014).

The cytokine hypothesis suggests that different environmental stressors as well as organic inflammatory conditions may trigger depression via inflammatory processes (Maes et al., 2009). Indeed, systemic infections, cancer or autoimmune diseases, as well as stressful life events, are characterized by an activation of the peripheral immune system, which is part of the required response of the body to cope with the adverse condition. However, when the activation of the immune system is prolonged, for example because of a persistence of the adverse event, cytokines and other immune modulators can access the brain and affect different brain systems that play a role in enhancing vulnerability to depressive disorders (Dantzer et al., 2008).

The neurotrophic hypothesis has been put forward based on a number of clinical and preclinical evidence suggesting that, beyond neurotransmitter changes, depression may be associated with structural abnormalities in different brain regions as well as defects in cell-cell communication (Frodl and O’Keane, 2013; Zhao et al., 2014). These alterations may be particularly relevant for core disease symptoms implying that therapeutic interventions should correct such defects in order to restore brain function in depressed subjects.

The goal of this review is to recapitulate the alterations in inflammation and neuronal plasticity that may be relevant for depression. Moreover, considering that the etiology of depression has been associated, at least in some individuals, with the exposure to stressful events early in life, we will discuss the possibility that alterations in inflammation-immune systems as well as dysfunction in neuronal plasticity may contribute to the long-lasting negative effects of stressful life events early in life and the consequent enhanced risk for depression.

## Depression and peripheral inflammation

There is strong evidence indicating that depression is associated with an activation of the innate immune system (Dantzer et al., 2008). This theory has been supported over the last 20 years by an increasing body of evidence showing alterations in the functional activity of the immune system in the blood and in the brain of depressed patients, as compared to control subjects (Kronfol et al., 1983; Maes, 1995; Maes et al., 1995a,b,c,d,e; Howren et al., 2009; Dowlati et al., 2010; Liu et al., 2012; Valkanova et al., 2013). To date, several studies have investigated blood and/or cerebrospinal fluid concentrations of one or more pro-inflammatory cytokines (e.g., interleukin IL-1β, IL-6, interferon gamma (IFN-γ)) and/or acute phase proteins (e.g., C reactive protein (CRP), an acute phase protein that promotes resistance to infection and repair of damages tissues) in depressed patients.

The majority of these studies, whose main results have been summarized in several meta-analyses (Howren et al., 2009; Dowlati et al., 2010; Liu et al., 2012; Valkanova et al., 2013) reported increased levels of IL-1β, IL-6, TNF-α and CRP in the serum and/or plasma of depressed patients. For example, Hestad et al. (2003) observed that subjects with depressive disorders had markedly increased TNF-α plasma levels compared with healthy controls and, similarly to TNF- α, also IL-6 plasma levels were increased in similar clinical samples (Sluzewska et al., 1996; Pike and Irwin, 2006). Changes of cytokine mRNA levels were also found when investigating peripheral blood cells. Indeed, Tsao and colleagues found higher mRNA levels of TNF-α, IL-1β, IL-6 and INF-α in the Peripheral Blood Mononuclear Cells (PBMCs) of patients suffering from MDD (Tsao et al., 2006), and our group has also shown an increased expression of cytokine mRNA levels in the leukocytes of drug free depressed patients as compared to controls (Cattaneo et al., 2013). Of note, the same cytokines have been significantly correlated with several clinical depressive “traits”. In particular higher cytokines levels have been associated with higher depression severity (Thomas et al., 2005) as well as with poor antidepressant response (Cattaneo et al., 2013; Powell et al., 2013; Stelzhammer et al., 2014). Similarly, CRP blood levels that, as mentioned above, are significantly elevated in depressed patients, may also represent a predictor of a poor outcome to antidepressant therapies (Danner et al., 2003; Ford and Erlinger, 2004; Ford et al., 2004; Howren et al., 2009; Pikhart et al., 2009; Uher et al., 2014).

Emerging evidence has proposed a role for cytokines also in child and adolescent depression (Mills et al., 2013), which is estimated to occur in approximately 2% of children and 4–8% of adolescents (Birmaher et al., 1996) and this may carry its own burden of disadvantages, often persisting or re-emerging at adulthood (Dunn and Goodyer, 2006; Weissman, 2009; Weissman and Talati, 2009). Moreover, similarly to adult depression, a de-regulation of the immune system, characterized by an imbalance between pro- and anti- inflammatory cytokines, has been observed in adolescent depression (Gabbay et al., 2009). To this regard, increased levels of pro-inflammatory cytokines, including IFN-γ, IL-6 and CRP, have been observed in depressed adolescents as compared to controls as well as in adolescents with a history of childhood trauma (Mills et al., 2013). Furthermore, the transition vs. depression development is accompanied by a further increase of these cytokines, which remain higher even after the depressive episode is improved (Miller and Cole, 2012).

Abnormalities in the immune and inflammatory systems occurring in depression are also found in post-mortem brains of depressed and suicide patients. Shelton and colleagues reported, for example, increased inflammatory pattern in the brain of depressed suicide patients (Shelton et al., 2011). Moreover, recent studies in the hypothalamus of depressed subjects have identified abnormalities in protein and mRNA levels of Toll Like Receptors (TLRs), which are involved in neuronal function as well as in the production of cytokines and chemokines in response to inflammation or stressful insults (Wang et al., 2008).

The role for inflammation in the pathogenesis of depression has been supported also by evidence showing that the administration of pro-inflammatory agents, like the endotoxin lipopolysaccharide (LPS), induces the development of depressive symptoms in humans (Grigoleit et al., 2011). In line with this, around the 30–40% of hepatitis C patients treated with the pro-inflammatory cytokine peg-interferon-alpha (pegIFN-α) develop clinically relevant depression (Miyaoka et al., 1999; Raison et al., 2005; Asnis and De La Garza, 2006). Finally, depression shows elevated comorbidity with several immune-related diseases, such as cancer, cardiovascular and neurodegenerative diseases, which are all clinical conditions characterized by the presence of inflammatory alterations (Benton et al., 2007; Anisman et al., 2008).

## Putative mechanisms underlying the association between depression and inflammation

**Figure 1:**
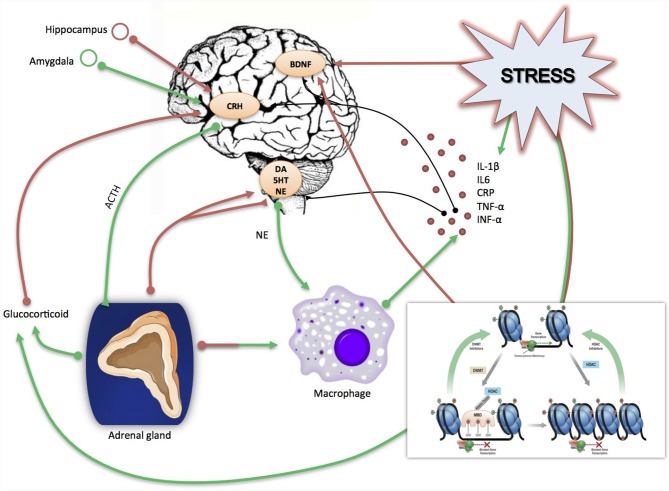
**Schematic rappresentation of the direct and indirect effect of stress on inflammation and neuroplasticity related processes**. Stress induces directly an immediate release of glucocorticoids and pro-inflammatory cytokines (IL-1β, IL-6, CRP, TNF-α, INF-α); in turn incresead levels of glucocorticoids act on the brain by altering the CRH-ACTH signaling and, in turn, negatively affecting neurogenesis as well as the production of neurotrophic factors, including Brain Derived neurotrophic Factor (BDNF). Similarly, proinflammatory cytokines can negatively affect brain functioning and neurotrophins production and release. Stress can also work indirectly by activating epigenetic mechanisms (methylation, deacetylation, miRNAs), which may act on the same target stress related genes i.e., glucocorticid receptors, cytokines and BDNF. Red arrows indicate a suppressive effect, green arrows a stimulating effect.

There are several mechanisms by which cytokines can access the brain, influence central neuronal functions and cause behavioral changes known as “sickness behavior”, a coordinated set of psychological and physiological modifications that develop during the course of an infection (Dantzer, 2004) and that resemble depressive symptoms. One pathway may involve macrophage-like cells located in the circumventricular organs and the choroid plexus, which detect and respond to circulating pathogen-associated molecular patterns by producing pro-inflammatory cytokines; these cytokines can then cross the Blood Brain Barrier (BBB) and affect neuronal function and microglia activation. Another mechanism by which cytokines can reach the brain is via binding with their specific transporters, which are located on the BBB. Moreover, microglia cells in the brain produce cytokines receptors and thus amplify the inflammatory signals (Besedovsky and del Rey, 1996; Capuron and Miller, 2004). Once in the brain, cytokines can affect brain function in a variety of ways, including the modulation of neurotransmitter metabolism and neurotoxic mechanisms. As an example, cytokines induce the enzyme Indoleamine 2,3 Dioxygenase (IDO), which breaks down the serotonin precursor tryptophan into kynurenine that, once converted into quinolinic acid, may lead to neurotoxicity through the activation of the glutamatergic system (Myint and Kim, 2014). Cytokines have also been shown to decrease the neurotrophic support and to reduce neurogenesis in several brain areas, particularly in the hippocampus (Hashmi et al., 2013; Williamson and Bilbo, 2013). This may eventually contribute to the reduction of neuronal plasticity that represents a core feature of depression-related dysfunction (see below). Furthermore, as we have also represented in Figure 1, cytokines can increase the levels of stress hormones, including corticotrophin releasing hormone (CRH), adreno-corticotrophin hormone (ACTH) and cortisol, which have been reported to be elevated in patients with depression (Besedovsky and del Rey, 1996; Pariante and Miller, 2001) and may therefore participate to HPA dysfunction (Miller et al., 2009).

Deregulation of microglia function has been associated with neurologic and psychiatric diseases and may lead to critical changes in neuronal activity and function (Beumer et al., 2012; Stertz et al., 2013; Paolicelli et al., 2014; Najjar and Pearlman, 2015).

One major mechanism through which microglia can alter brain functions associated with psychiatric diseases is neurogenesis. The impact of inflammation on adult hippocampal neurogenesis was originally discovered by the groups of Lindvall and Palmer, demonstrating that systemic or intra-hippocampal administration of LPS reduces the formation of newborn neurons in the adult hippocampus, an effect that can be prevented by indomethacin, a non steroidal anti-inflammatory drug, which act by inhibiting the synthesis of pro-inflammatory prostaglandins (Ekdahl et al., 2003; Monje et al., 2003).

Microglia can exert a positive or negative influence on proliferation, survival, or differentiation of newborn cells, depending on the inflammatory context. For instance, microglia can compromise the neurogenic cascade during chronic stress, through the release of pro-inflammatory cytokines such as IL-1β, IL-6, and TNF-α. Microglia has been also shown to phagocyte the excess of newborn neurons undergoing apoptosis in the hippocampal neurogenic niche during normal physiological conditions, while a similar role in the synaptic integration of newborn cells was also proposed in light of microglial cells to phagocyte synaptic elements (Sierra et al., 2014). Kreisel et al. provided also a link between stress-induced alterations in microglia and the development of stress-induced depression (Kreisel et al., 2014). Indeed they showed a role of dynamic alterations in microglia activation status in the development of chronic unpredictable stress (CUS)-induced depressive-like condition in rodents and the ability of minocycline and of the transgenic interleukin-1 receptor antagonist to rescue the subsequent microglial apoptosis, as well as the CUS-induced depressive-like behavior and suppressed neurogenesis.

It has to be mentioned that depending on its activation state, microglia may have opposite effects on adult neurogenesis and it is likely that pro-neurogenic and anti-neurogenic microglial cells may co-exist, with a different responsiveness to external stimulus, such as voluntary running and housing conditions. Thus it may be inferred that the overall impact on adult neurogenesis may depend on the outcome of the interaction between environmental factors and microglial state (Gebara et al., 2013).

Cytokines alterations in depression have also important implications with respect to the response to pharmacological treatments. On one end, different studies have demonstrated the ability of some antidepressants to reduce cytokines activation in depressed patients (Sluzewska et al., 1995; Frommberger et al., 1997; Tuglu et al., 2003; Basterzi et al., 2005). Our research group has recently demonstrated that cytokine expression in the leukocytes from depressed patients are reduced following escitalopram and nortriptyline treatment with a significant correlation between these changes and treatment response (Cattaneo et al., 2013).

Moreover, depressed patients who are non responders to antidepressant therapies or who are treatment resistant show higher plasma concentrations of several pro-inflammatory cytokines and CRP as compared to responders (Sluzewska et al., 1997; Lanquillon et al., 2000; Fitzgerald et al., 2006; Uher et al., 2014). In line with these results, we found that patients who do not respond to two different classes of antidepressants have higher baseline mRNA levels of IL-1β, macrophage migration inhibitory factor (MIF), and TNF-α (Cattaneo et al., 2013). Similar results on the role of TNF-α in treatment response were also reported by Powell et al. (2013).

It may be argued that peripheral inflammation could alter behavioral response to monoaminergic drugs because high levels of cytokines are known to modulate monoamine synthesis, reuptake and metabolism, for example by altering the function of the serotonin transporter, which is a key target of antidepressant drugs (Tynan et al., 2012). Thus, cytokine-induced changes in monoaminergic signaling may not only induce depressive states, but may conceivably compromise the therapeutic effects of monoamine reuptake inhibitors, leading to first-line treatment resistance. Conversely, monoaminergic drugs may impact directly the inflammatory gene expression or peripheral immune cells, although this possibility has yet to be fully tested and established (Pollak and Yirmiya, 2002).

## Depression and neuronal plasticity

Neuronal plasticity is a concept that refers to a number of mechanisms crucial for brain function and its ability to perceive, adapt and respond to a variety of internal and external stimuli. It is thought that such mechanisms can be defective in different psychiatric disorders and this may eventually enhance disease susceptibility (Manji et al., 2003; de Kloet et al., 2005; Duman and Monteggia, 2006; Calabrese et al., 2009).

A large body of evidence has demonstrated that stress, a major environmental challenge for depression, can lead to an impairment of neuronal plasticity (McEwen et al., 2012; Bohacek et al., 2014). Among the systems contributing to the maintenance of neuronal plasticity, neurotrophic factors, and in particular the neurotrophin Brain-Derived Neurotrophic Factor (BDNF), have emerged as important mediators for long-term functional deterioration associated with mental illness (Bramham and Messaoudi, 2005; Lu et al., 2005; Duman and Monteggia, 2006; McClung and Nestler, 2008; Cirulli et al., 2009; Castrén and Rantamäki, 2010a; Calabrese et al., 2011b; Chourbaji et al., 2011). BDNF, in fact, is not only important during brain development, but it exerts a pivotal role for neuronal remodeling as well as synaptic function (Lu et al., 2008; Waterhouse and Xu, 2009). Several studies have demonstrated that, in depressed subjects, the expression of BDNF is reduced in brain structures, such as the hippocampus and the prefrontal cortex, which represent key anatomical targets for stress-induced structural changes. Preclinical studies have confirmed the association between stress exposure and BDNF, since chronic exposure to different stress paradigms leads to a consistent reduction of neurotrophin expression (Pittenger and Duman, 2008) (Tsankova et al., 2006). The expression of BDNF is also reduced in the hippocampus and prefrontal cortex of serotonin transporter knockout rats, a genetic model of depression and anxiety (Molteni et al., 2010), suggesting that changes of neuronal plasticity may also contribute to the genetic susceptibility to mood disorders.

Changes of BDNF expression may represent a relevant component for functional disability. For example it has been shown that targeted or inducible deletion of the BDNF gene produces behavioral dysfunction related to anxiety and depression (Chourbaji et al., 2011; Burke et al., 2013), suggesting that such changes may contribute to the pathologic condition. Furthermore BDNF expression plays a critical role in resilience to chronic stress and in the development of neural circuits that control coping mechanisms (Taliaz et al., 2011).

Since the expression of trophic factors is reduced in depression and this may contribute to functional defects associated with the pathologic condition, it may be inferred that effective pharmacological intervention should be able to normalize such alterations. Indeed, a key step in long-term adaptive changes brought about by antidepressants appears to be their ability to modulate the expression of BDNF as well as of other growth factors (Berton and Nestler, 2006; Groves, 2007; Martinowich et al., 2007; Calabrese et al., 2009, 2011a; Castrén and Rantamäki, 2010b; Cattaneo et al., 2013). The majority of the studies focusing on BDNF have demonstrated that these drugs can modulate neurotrophin transcription (Coppell et al., 2003; Molteni et al., 2006; Calabrese et al., 2007, 2011a; Nair et al., 2007; Kozisek et al., 2008), its translation and trafficking to specific sub-cellular compartments (Calabrese et al., 2007), as well as BDNF receptor activation and signaling (Saarelainen et al., 2003; Fumagalli et al., 2005; Duman et al., 2007). The ability to modulate BDNF has also been demonstrated for the rapid acting antidepressant ketamine (Autry et al., 2011). A number of experimental studies have shown that defective BDNF expression or release may limit the antidepressant activity (Wolkowitz et al., 2011; Dreimüller et al., 2012), suggesting that neurotrophin modulation may represent an important mechanism of antidepressant drugs. This possibility is in accordance with clinical data demonstrating that serum BDNF levels, which are reduced in depressed subjects, can be normalized only in patients that are responsive to pharmacological intervention (Bocchio-Chiavetto et al., 2010; Yoshida et al., 2012; Molendijk et al., 2014).

The modulation of neurotrophic proteins can lead to functional and structural changes affecting brain regions key to depressive symptoms. One of the mechanisms that lie downstream from neurotrophic factors is neurogenesis, the process by which neurons are generated from stem cells. Indeed chronic antidepressant treatment can increase neurogenesis in the adult brain, primarily in the subgranular zone of hippocampal dentate gyrus, a mechanism that depends on the modulation of trophic factors and that appears to be relevant for the behavioral action of antidepressant drugs (Cameron et al., 1998; Duman et al., 2001; Santarelli et al., 2003; Malberg, 2004; Sairanen et al., 2005; Banasr and Duman, 2008).

## Childhood trauma as vulnerability factor for depressive phenotypes

A recent European Report from WHO indicates that at least 18 million children in Europe suffer from early life trauma, harming mental and physical health, and with enormous societal costs, including for medical and social care (Europe WHO of European report on preventing child maltreatment).

Childhood maltreatment is defined as acts of commission or omission by parents or caregivers resulting in potential harm to the child’s health, and includes experiences such as physical, sexual and psychological abuse, as well as physical or emotional neglect. Among substantiated reports, 60% of the childhood maltreatment is classified as neglect, 20% as physical abuse, and 10% as sexual abuse (Holmes and Slap, 1998). The prevalence of emotional abuse and neglect is likely much higher than that of sexual and physical abuse, but more difficult to measure and quantify (Holmes and Slap, 1998).

A number of studies have shown that the onset of mood disorders, such as depression, is undoubtedly influenced by stressful life events that occur in childhood (Kendler et al., 2004a,b; Horesh et al., 2008). In one community-based study of approximately 2,000 women, those with a history of childhood physical or sexual abuse had an increased risk of depression and anxiety and were more likely to have attempted suicide than women without such a history (Kendler et al., 2004a,b). It is also evident that different types of child maltreatment have long-term adverse consequences for mental health (Cicchetti and Toth, 2005; Gonzalez, 2013; Allen et al., 2014; Bailer et al., 2014; Cummings and Berkowitz, 2014; Hagan et al., 2014; Roth et al., 2014). Among the different types of maltreatment, sexual abuse is probably the most relevant with respect to increased risk for psychiatric disorders, such as depression and anxiety (Booth and Gulati, 2014; Kanamüller et al., 2014; Letourneau et al., 2014; Visser et al., 2014). On these bases, there is high interest in understanding, which are the mechanisms that may link the exposure to adversities early in life with the enhanced susceptibility to mood disorders.

## Childhood trauma and alterations in the inflammatory system

Although the association between early life stressful events and depression may occur via several biological processes, a number of studies have suggested a role for increased inflammation or increased sensitivity of inflammatory responses. Taking advantage of the Dunedin cohort subjects, Danese et al. were the first to demonstrate that elevated CRP blood levels were significantly associated with maltreatment during childhood (Danese et al., 2008) and such association was particular strong in individuals that developed depression later in life (Danese et al., 2008, 2009). Similarly Slopen et al. reported that exposure to childhood adversities is associated with higher levels of IL-6 and CRP in teenagers (Slopen et al., 2014).

It has also been shown that depressed subjects with a history of early life stress show an increased inflammatory response when re-exposed to an acute psychological stress at adulthood, as indicated by an exaggerated IL-6 response as well as increased DNA binding of the key pro-inflammatory transcriptio factor, nuclear factor kappa-light-chain-enhancer of activated B cells (NF-κB) in PBMCs (Pace et al., 2006).

Based on this evidence, it is possible to speculate that individuals who experience major stressors early in life are more vulnerable to an immune dysregulation at adulthood, regardless of whether they subsequent develop adverse physical or mental health consequences. Miller and Chen have proposed a model suggesting that stress that occurs during a sensitive period in life, when immune function is highly plastic, gets embedded in the functioning of the cells that regulate inflammation (Miller and Chen, 2007). Therefore, brain inflammatory cells including macrophages, microglia and dendritic cells, will develop a hyper-sensitivity that leads to a chronic pro-inflammatory state, due to an activation of pro-inflammatory transcription factors such as NF-κB and down-regulation of anti-inflammatory transcriptions factors such as the glucocorticoid receptor, thus increasing the levels of circulating cytokines. In addition, an altered response of innate immune cells to stimuli causes abnormalities in other leucocytes, particularly the T- and B- cells that orchestrate adaptive immune responses.

How childhood trauma generates a “pro-inflammatory” phenotype is still an open question but it is probably the result of a deregulation in complex networks within biological pathways affected by such experiences (see Figure 1). With this respect, the study of epigenetic processes holds a substantial promise to explain many of these unsolved questions, since epigenetic operates at the interface between the individual genetic background and the environment.

Studies in rodents have also shown that early life stress induces a premature activation of the immune system that can significantly shift the developmental trajectory of microglia, changing the long-term patterns of activation of these cells (Schwarz et al., 2011; Williamson et al., 2011). As a consequence of these changes, rats exposed to stress early in life are more vulnerable to increase in pro-inflammatory cytokines production following an LPS challenge in the adulthood, suggesting that this pro-inflammatory state persists in time and can be responsible of an enhanced vulnerability and sensitivity to a novel insult in adulthood (Sominsky et al., 2013).

## Early life adversities and long-term changes in neuronal plasticity

Since neuronal plasticity may contribute to structural modifications and to the inability to respond or adapt to environmental challenges (Berton and Nestler, 2006; Krishnan and Nestler, 2008; McClung and Nestler, 2008; Pittenger and Duman, 2008; Calabrese et al., 2009), it is feasible to hypothesize that alterations of these mechanisms may also represent the long-lasting consequence of stressful experience occurring early in life.

In accordance with this possibility, a long-term reduction of BDNF expression and function may represent a common endpoint for adverse experience early in life, although the anatomical specificity of such changes depends on the type, timing and duration of the manipulation. Indeed, BDNF mRNA levels are reduced in the hippocampus of adult rats that were exposed to 24 h of maternal deprivation at postnatal day 9 (Roceri et al., 2002), whereas more protracted manipulations during gestation or the early phase of postnatal life (such as prenatal stress or repeated maternal deprivation) reduce the levels of the neurotrophin, primarily in the prefrontal cortex (Koo et al., 2003; Fumagalli et al., 2004; Roceri et al., 2004; Roth et al., 2009). The time course analysis of BDNF changes in rats exposed to prenatal stress (PNS) suggests that the reduced expression observed in adult animals is not directly linked to stress exposure, but is dependent on the maturational stage of the prefrontal cortex, becoming fully manifest after adolescence (Luoni et al., 2014). Moreover we have recently demonstrated that exposure to PNS leads to a significant down-regulation of the pool of BDNF transcripts with long 3’UTR that are responsible for targeting BDNF mRNA to dendrites, where activity-dependent translation may occur (An et al., 2008; Lau et al., 2010). Hence, the selective decrease of long 3’UTR BDNF mRNA levels after PNS may contribute to defects in local, activity-dependent neurotrophin synthesis (Lau et al., 2010), which may eventually lead to reduced cell-cell communication and synaptic function and ultimately contribute to cognitive and emotional deterioration associated with exposure to early life adversities (Murmu et al., 2006; Michelsen et al., 2007). Interestingly, reduced neurogenesis was also found in response to stress early in life. For example, PNS in rats induced lifespan reduction of neurogenesis in the dentate gyrus and leads to an impairment of hippocampal-related spatial tasks (Lemaire et al., 2000). Similar stressful experiences in monkeys can result in reduced hippocampal volume and an inhibition of neurogenesis in the dentate gyrus, which is associated with increased pituitary-adrenal activity, as well as with behavioral profiles indicative of greater emotionality (Coe et al., 2003). Furthermore, it has been demonstrated that the exposure to prolonged, but not brief, bouts of maternal separation during the first 2 weeks of life determines a long-lasting suppression of adult neurogenesis and diminished plasticity in this parameter after exposure to stress in adulthood (Mirescu et al., 2004). Interestingly, some of the neuroplastic alterations brought about by early life stress can be normalized or even prevented by pharmacological intervention during early life, adolescence as well as adulthood (Matrisciano et al., 2012; Luoni et al., 2014).

## Childhood trauma, inflammation and depression: is epigenetic the linking mechanism?

The term “epigenetics” refers to long-lasting changes in gene expression without alterations of the DNA sequence, which are associated with several potentially reversible processes including DNA methylation, histone modifications and aberrant expression of micro-ribonucleic acid (miRNA; Maffioletti et al., 2014; Provençal and Binder, 2014a). Among different epigenetic modifications, DNA methylation is one of the best-characterized mechanisms in relation to childhood adversities (Essex et al., 2013). Indeed changes of DNA methylation at sensitive gene promoters may explain the persistence of early life effects into adulthood, rendering the subject more vulnerable and sensitive to subsequent insults and challenges.

In humans, DNA methylation occurs, almost exclusively, through covalent modification of DNA, where methyl groups are coupled to cytosine residues of CpG dinucleotides. DNA methylation has been shown to be associated with variations in gene expression (Szyf, 2013; Reul, 2014), thus serving as a possible mechanism for regulating the transcriptional response to extracellular events. Several preclinical studies have highlighted how exposure to environmental stressors can produce long-lasting behavioral alterations and may affect coping abilities later in life through epigenetic modifications and in particular through changes in DNA methylation within selected brain regions (Szyf and Bick, 2013; Provençal and Binder, 2014a; Booij et al., 2015; Desplats, 2015). For example, in rats, reduced maternal care produces long lasting effects on the offspring, including an anxious phenotype and higher corticosterone levels in response to stress. These behavioral abnormalities are associated with reduced hippocampal expression of glucocorticoid receptors that appears to be the consequence of increased methylation at gene promoter (Meaney and Szyf, 2005; Szyf et al., 2005; Kofink et al., 2013). Also, maternal separation in mice is able to induce an hypomethylation in the vasopressin gene enhancer region, which leads to increased expression of hypothalamic vasopressin, accompanied by enhanced corticosterone secretion (Murgatroyd et al., 2009). Some of these changes have been shown to occur also in humans. Indeed, McGowan et al. have demonstrated that in human post-mortem brain studies early life abuse was associated with increased methylation of the GR exon 1f promoter in the hippocampus, in support of the “translational” implications for the epigenetic changes brought about by the exposure to early life stress (McGowan et al., 2009). In addition to the stress-responsive systems, also neuroplastic genes can undergo epigenetic regulation, which may be responsible for the changes observed in mental illness. At experimental level, it was demonstrated that the persistent reduction of BDNF expression in the social defeat stress paradigm is due to epigenetic changes in the promoter region of two of its transcripts (Tsankova et al., 2006). Similarly, we have recently shown that the expression of BDNF is significantly reduced in the prefrontal cortex of serotonin transporter knockout rats through an increased methylation in the promoter region of exons VI and reduced H3 acetylation at exon IV (Molteni et al., 2010). These results are in line with post mortem studies since increased BDNF promoter methylation has been found in the brain of suicide subjects (Keller et al., 2010). Such modification may also represent the consequence of early life adversities. Indeed, maltreatment during infancy in rodents produces a persistent increase of the methylation in BDNF exon-4 and exon-9 that leads to reduced neurotrophin expression in the adult prefrontal cortex (Roth et al., 2009).

A growing number of studies is addressing the consequences of early life stress on DNA methylation at genome wide level in the brain as well as in peripheral tissues (Mehta et al., 2013; Nieratschker et al., 2014; Provençal and Binder, 2014a) in order to identify signatures that may be associated with the long-term pathologic consequences of such experiences. With this respect epigenetic changes in peripheral tissues may correlate to some extent with measures in the brain. As an example differential rearing conditions of rhesus macaques is associated with differential methylation in early adulthood in both the brain and T cells, suggesting that the response to early-life adversity is system-wide and genome-wide and persists to adulthood (Provençal et al., 2012). Furthermore the observation that ELS-associated DNA methylation changes are not limited to the brain but can be found in peripheral systems suggests that such changes may also be relevant for additional health problems, such as the described increased risk for cardiovascular and metabolic diseases (Provencal and Binder, 2014b).

With this respect it will be extremely important to investigate and characterize inflammatory-immune methylation signatures as a consequence of early life stress, which will eventually provide key information not only for their role in mental illness but also as a potential mechanism to explain the comorbidity of depression with different medical conditions.

## Conclusions

As discussed in this review, there is evidence linking early life stressful events, peripheral inflammation, alterations in neuroplastic mechanisms and depression, although the underlying biological mechanisms still need to be clarified. We have discussed the role of epigenetics, and in particular of DNA methylation, as one such mechanism. Indeed, early life stressful events can activate epigenetic mechanisms at global levels as well as at the promoter regions of key target genes, producing long-lasting and stable changes in gene expression, which persist up to adulthood and may be responsible of an increased vulnerability to develop mental disorders. Through a better understanding of how epigenetic mechanisms underlie psychiatric disorders, we could also better characterize how these modifications can have an impact on specific genes that, in turn, contribute to the pathogenesis of these disorders. Moreover, as increased inflammation is clearly observed in depressed patients and, in particular, in those do not respond to antidepressant therapies, future research will aim to clarify whether increased inflammation actually identifies a single group of depressed patients that has experienced childhood maltreatment and is also resistant to conventional antidepressants. Moreover, inflammatory biomarkers may be used as strategy to screen patients who may benefit from drugs that target inflammatory mechanisms. Finally, future studies should also provide new insights on the reversibility of the damage associated with childhood stress experiences, including studies testing whether pharmacological and non-pharmacological interventions could reverse the abnormalities induced by childhood adversities on the functionality of the immune and stress response systems and thus also minimize the risk for mood disorders, both in the individuals affected and in the next generations.

## Authors statement

AC designed the work and performed most of the literature work; she achieved the first draft of the paper; she approved the final version of the manuscript and she agreed to have accounted for all the aspects of the work. FM performed the literature work and contributed to the interpretation of the data; she drafted the manuscript, approved the final version of the manuscript and she ensured that all the aspect of the work have been appropriately investigated. GP contributed to the conception of the work and to draft the manuscript; he approved the final version of the manuscript and agreed to have accounted for all the aspects of the work. VB contributed substantially to the revised version of the manuscript and to the data interpretation; she drafted of the manuscript; she approved the final version of the manuscript and she ensured that all the aspects of the work have been appropriately investigated.

LBC contributed to the interpretation of data for the work; she revised critically the manuscript, and she ensured that all the aspects of the work have been appropriately investigated.

MAR contributed substantially to the conception of the work, he revised the manuscript critically for important intellectual content, he approved the final version of the manuscript and agreed to have accounted for all the aspects of the work.

CMP contributed substantially to the conception of the work and to the data discussion; he revised the manuscript critically for important intellectual content, he approved the final version of the manuscript and agreed to have accounted for all the aspects of the work.

All the authors approve and confirm their role in the manuscript and also the order in which they do appear.

## Conflict of interest statement

The authors declare that the research was conducted in the absence of any commercial or financial relationships that could be construed as a potential conflict of interest.
